# Implementation and resource needs for long‐acting PrEP in low‐ and middle‐income countries: a scoping review

**DOI:** 10.1002/jia2.26110

**Published:** 2023-07-13

**Authors:** Delivette Castor, Craig J. Heck, Daniela Quigee, Niharika Vasant Telrandhe, Kiran Kui, Jiaxin Wu, Elizabeth Glickson, Kibret Yohannes, Sergio Torres Rueda, Fiammetta Bozzani, Kathrine Meyers, Jason Zucker, Justine Deacon, Katharine Kripke, Magdalena E. Sobieszczyk, Fern Terris‐Prestholt, Christine Malati, Chris Obermeyer, Anita Dam, Katie Schwartz, Steven Forsythe

**Affiliations:** ^1^ Division of Infectious Diseases Columbia University Irving Medical Center New York New York USA; ^2^ Department of Epidemiology Columbia University Mailman School of Public Health New York New York USA; ^3^ New York Medical College Valhalla New York USA; ^4^ University of Virginia School of Medicine Charlottesville Virginia USA; ^5^ The London School of Hygiene and Tropical Medicine London UK; ^6^ The Aaron Diamond AIDS Research Center Columbia University Irving Medical Center New York New York USA; ^7^ The CDC Foundation Atlanta Georgia USA; ^8^ Avenir Health Takoma Park Maryland USA; ^9^ United States Agency for International Development Washington DC USA; ^10^ The Global Fund to Fight AIDS, Tuberculosis and Malaria Geneva Switzerland; ^11^ FHI360 Durham North Carolina USA; ^12^ Avenir Health Glastonbury Connecticut USA

**Keywords:** economics, healthcare costs, implementation planning, LMICs, long‐acting HIV prevention, pre‐exposure prophylaxis

## Abstract

**Introduction:**

Several low‐ and middle‐income countries (LMICs) are preparing to introduce long‐acting pre‐exposure prophylaxis (LAP). Amid multiple pre‐exposure prophylaxis (PrEP) options and constrained funding, decision‐makers could benefit from systematic implementation planning and aligned costs. We reviewed national costed implementation plans (CIPs) to describe relevant implementation inputs and activities (domains) for informing the costed rollout of LAP. We assessed how primary costing evidence aligned with those domains.

**Methods:**

We conducted a rapid review of CIPs for oral PrEP and family planning (FP) to develop a consensus of implementation domains, and a scoping review across nine electronic databases for publications on PrEP costing in LMICs between January 2010 and June 2022. We extracted cost data and assessed alignment with the implementation domains and the Global Health Costing Consortium principles.

**Results:**

We identified 15 implementation domains from four national PrEP plans and FP‐CIP template; only six were in all sources. We included 66 full‐text manuscripts, 10 reported LAP, 13 (20%) were primary cost studies‐representing seven countries, and none of the 13 included LAP. The 13 primary cost studies included PrEP commodities (*n* = 12), human resources (*n* = 11), indirect costs (*n* = 11), other commodities (*n* = 10), demand creation (*n* = 9) and counselling (*n* = 9). Few studies costed integration into non‐HIV services (*n* = 5), above site costs (*n* = 3), supply chains and logistics (*n* = 3) or policy and planning (*n* = 2), and none included the costs of target setting, health information system adaptations or implementation research. Cost units and outcomes were variable (e.g. average per person‐year).

**Discussion:**

LAP planning will require updating HIV prevention policies, technical assistance for logistical and clinical support, expanding beyond HIV platforms, setting PrEP achievement targets overall and disaggregated by method, extensive supply chain and logistics planning and support, as well as updating health information systems to monitor multiple PrEP methods with different visit schedules. The 15 implementation domains were variable in reviewed studies. PrEP primary cost and budget data are necessary for new product introduction and should match implementation plans with financing.

**Conclusions:**

As PrEP services expand to include LAP, decision‐makers need a framework, tools and a process to support countries in planning the systematic rollout and costing for LAP.

## 1 INTRODUCTION

Of the nearly 1.5 million annual new HIV acquisitions globally, most occur in low‐ and middle‐income countries (LMICs), and 70% of acquisitions occur in sub‐Saharan Africa (SSA) [1, 2, 3]. In 2020, most of the 14 countries that achieved UNAIDS “Fast‐Track” targets of 73% across the testing and treatment cascade were LMICs, and seven were in eastern and southern Africa (ESA) [4] In countries achieving UNAIDS Fast‐Track targets, new HIV acquisitions have declined from 39% in Uganda to 70% in Zimbabwe, but no country has achieved the projected elimination target [1, 2]. Fast‐track goals for comprehensive HIV prevention, including 3 million person‐years of pre‐exposure prophylaxis (PrEP) by 2020, were not achieved [5]. Additionally, more than two in five oral PrEP users globally discontinue within 6 months of initiation with higher rates in LMICs and women [6]. PrEP discontinuation rates may represent individual‐level changes in potential HIV exposure or it may signal a preference for products other than daily oral PrEP [7].

In July 2022, the World Health Organization (WHO) added conditional recommendations for two long‐acting HIV prevention interventions: the monthly dapivirine vaginal ring (PrEP ring) and long‐acting cabotegravir (CAB LA; injectable PrEP), joining oral PrEP as part of combination HIV prevention [8, 9]. The 2022 WHO Guidelines mark an unprecedented moment in HIV prevention, when multiple PrEP methods are recommended as part of biomedical prevention for all at elevated risk of HIV (Appendix S1) [8, 9, 10, 11, 12, 13, 14, 15, 16, 17, 18]. Notably, for the first time, cis‐gender women can utilize three HIV PrEP methods: daily oral PrEP, PrEP ring, and injectable PrEP [10, 11, 15]. By October 2022, the monthly PrEP ring had been approved in six countries in ESA and is currently undergoing regulatory review in six additional ESA countries [19]. Long‐acting cabotegravir received approval from the US Food and Drug Administration and the Australian Therapeutic Goods Administration in December 2021 [20] and August 2022, respectively. In October 2022, Zimbabwe became the first LMIC to approve injectable PrEP for use [21, 22]. Injectable PrEP has also been approved by South African Health Products and Regulatory Authority (SAPHRA) and is currently undergoing regulatory review in several LMICs [19, 21].

Evidence review of “Fast‐Track” goals highlighted global challenges in implementation and widening funding gaps that reduced the impact of HIV prevention amid biomedical advancements [4]. The UNAIDS strategy 2021–2026 highlights the need to systematize HIV prevention implementation and address the widening funding gap for the HIV response, particularly financing for HIV prevention [3]. In an earlier review of daily oral PrEP costs and cost‐effectiveness modelling studies, Case et al. highlighted the poor quality of PrEP cost‐effectiveness and modelling studies and the lack of primary cost data collection, “real world costs,” or inclusion of service delivery strategies in modelling studies [23, 24]. The introduction long‐acting pre‐exposure prophylaxis (LAP) marks an opportunity for choice in HIV prevention, accompanied by increased complexity in health service delivery. This opportunity should be met with plans to guide and assess implementation systematically. Further, improved cost analyses are critical to inform such plans [1, 3]. Without these, the recognized deficiencies threaten to deepen and jeopardize LAP's potential. With this scoping review, we sought to inform costed plans for LAP implementation by: (1) collating and synthesizing evidence from costed national plans of oral PrEP and family planning (FP) implementation; (2) developing a consensus on the range of key activities and inputs needed for systematic delivery of PrEP innovations, including LAP (hereafter referred to as implementation domains); (3) appraise the cost evidence that would typically inform national implementation plans using this implementation framework; and (4) provide recommendations on future considerations for improving systematic LAP delivery.

## 2 METHODS

### 2.1 Defining implementation domains

We reviewed publicly available national costed PrEP implementation plans to identify implementation domains that will help achieve national PrEP scale‐up or impact goals and objectives. Broad searches were conducted through Google, and focused searches were conducted through websites of national ministries of health, multilateral agencies and digital repositories, like PrEPwatch.com. Implementation details were extracted and mapped to describe the real‐world consensus of implementation domains. We also mapped domains from templates of FP costed implementation plans (CIPs) [25, 26, 27, 28].

### 2.2 Search strategy

We conducted a scoping review of PrEP costing and cost‐effectiveness studies adhering to the Cochrane Handbook 5.1 and Preferred Reporting Items for Systematic reviews and Meta‐Analyses extension for Scoping Reviews (PRISMA) scoping review guidance [29] (Supporting Information S2). Between 1–30 June 2022, we searched nine databases for peer‐reviewed literature: PubMed, Medline, Web of Science, Embase, PsychInfo, Africa Wide Information, Global Index Medicus, Cochrane and Econlit—using terms regarding (1) PrEP methods, (2) costing and (3) LMICs to identify potential publications (Supporting Information S3). Lastly, we solicited the International AIDS Economics Network for unpublished reports and non‐peer‐reviewed literature.

### 2.3 Inclusion criteria

We included all studies reporting PrEP interventions currently or imminently available (e.g. daily oral, event‐driven PrEP, PrEP ring, and CAB LA), or likely to make a market debut within the next 10 years (see Supporting Information S3 for full list) [30]. Additional inclusion criteria were that the study: (1) measured cost or estimated cost through primary data collection or other methods, including epidemiologic and mathematical modelling; (2) was published between 1 January 2010 and 30 June 2022; (3) reported cost data (e.g. average or incremental) or economic evaluation outcomes, such as cost‐effectiveness, cost‐benefit, or cost‐utility; (4) was conducted in any LMIC (classified using World Bank categorizations [31]); and (5) was published in English. We compared our final study sample with other recent reviews on the modelling and cost‐effectiveness of biomedical HIV prevention by Bozzani et al. and Giddings et al. [32, 33]. We chose 2010 because clinical trials for daily oral PrEP were underway, and many countries were already considering ways to incorporate PrEP into national HIV plans, pending proven safety and efficacy. We excluded publications reporting only qualitative data; assessing treatment as prevention, microbicides, vaccines, or broadly neutralizing antibodies only; examining high‐income country settings only; missing full texts; and conveying aggregate (other reviews), subjective (letters to the editor, commentaries), formative (study protocols), or theoretical (not reporting cost or cost‐effectiveness) research information.

### 2.4 Data screening and extraction

Two reviewers (EG and NT) independently screened titles and abstracts for eligibility using Covidence (Veritas Health Innovation, Australia), followed by a review of full‐text articles. Three investigators (DC, SF and CJH) independently reviewed and resolved discrepant screenings and reviewed a sub‐sample of concordant studies to validate the agreement. When needed, the broader team discussed and resolved discrepancies. Co‐authors (DC, SF, CJH, NT, JW and KK) extracted relevant data from the included studies into REDCap [34]. Co‐authors (FB, FTP and STR) conducted an independent review to supplement this review's findings [32, 35].

The data collection instrument included: implementation domains previously identified, author, country, year of publication, study year, study purpose, study design, population(s), intervention(s), perspective, duration of observation, period type, sampling strategies, data collection, scope, cost type, estimation method of inputs, discount and inflation rates, analytic methods and findings, transparency regarding limitations, conflicts of interest, and data availability. Data abstraction was disaggregated by geographic area, priority population, PrEP method, and service delivery platform for each cost or economic result. For articles published before 2015, we classified reports of PrEP as daily oral PrEP if the authors did not explicitly state the type of PrEP method.

### 2.5 Assessment of financial and economic evidence

We utilized principles 1–17 from the Global Health Costing Consortium (GHCC) reference case, which presents principles of quality and completeness of costing studies of health interventions with qualitative and quantitative information [25, 26, 27, 28, 36].

### 2.6 Data analysis and synthesis

We assessed inter‐rater reliability using Cohen's Kappa (*K*), a statistic that accounts for agreement due to random chance, at each screening stage [37, 38]. We created a PRISMA diagram to present the number of included and excluded publications [39]. Descriptive statistics were used to summarize the study characteristics from quantitative variables (i.e. frequencies and percentages for categorical variables; maximums, medians and modes for continuous variables). We synthesized extracted texts on costs and assumptions. Given the diversity of study designs included in the review, we did not conduct assessments of quality or bias.

## 3 RESULTS

### 3.1 National costed PrEP implementation plans

Our search for available costed national PrEP operational plans from LMICs yielded four country plans from ESA: Kenya, South Africa, Zambia and Zimbabwe [25, 26, 27, 28]. No publicly available plans included LAP.

### 3.2 Identified implementation domains

Also, we examined the GHCC and the template for developing CIPs for FP, as well as some resulting national CIPs, as source files for identifying implementation domains [36, 40, 41]. Reviewing these reports, we identified the following 15 key inputs and activities (implementation domains) in at least one document (Table 1) that represent our study team's consensus:

1. **National coordination, policy and planning**—Leadership, governance, and activities to increase ownership and coordination of the HIV PrEP response. Additional activities include implementation planning, adaptation and dissemination of guidelines and policies, community and stakeholders’ engagement, and coordination to start up, scale, or sustain PrEP programme.
2. **Target setting**—Activities to define priority populations, coverage levels, the pace of introduction and scale‐up, and other rollout scenarios to achieve impact.
3. **Communication/awareness raising/demand creation**—Activities to increase knowledge and awareness of PrEP services, create demand for PrEP among priority populations and PrEP advocacy at all levels.
4. **Service delivery approaches**—Includes service entry points for integrating PrEP, and feasibility of integrating PrEP into other services. We particularly focused on non‐HIV services integration, such as FP, sexual and reproductive health, antenatal care, and community‐based services. We also identified inclusion into HIV programmes, including self‐testing.
5. **Counselling and adherence support**—Includes counselling to initiate, sustain, discontinue, and adhere to PrEP.
6. **Human resources**—Includes in‐service and pre‐service education and training for physicians and allied health professionals.
7. **PrEP intervention (commodities)**—Includes cost of PrEP products.
8. **Laboratory monitoring services and other commodities**—Includes baseline tests for eligibility and safety monitoring.
9. **Supply chain management and logistics**—Includes commodity inventory management, reporting, tracking, and handling procedures for distributing PrEP to service delivery points. This includes warehousing and distribution of PrEP and other necessary commodities.
10. **Health information systems**—Developing and updating information systems and registers to document and report PrEP services for quality improvement or reporting purposes.
11. **Monitoring and evaluation**—Activities to define indicators, include PrEP monitoring as part of routine HIV services, and continuous quality control and improvement to ensure that the services are of the highest possible standard.
12. **Implementation science and operations research—**Includes planned research activities to facilitate and inform scale‐up.
13. **Budgeting, costing and financing**—Budget, cost, and economic evaluations to develop cost estimates of PrEP service delivery, impact, and financing shortfalls. This includes stakeholder engagement to blend finances through public–private partnerships to support PrEP delivery.
14. **Indirect/overhead**—Includes costs that cannot be directly traced to the provision of a service, such as administration, security personnel, buildings, and general equipment.
15. **Above‐site activities**—Includes various support services provided by the central administration, such as training, education and outreach, demand generation campaigns, central laboratory services, technical assistance, and capacity building.

**Table 1:** Mapping of key implementation domains drawn from costed national implementation plans for daily oral PrEP.

Kenya: Framework for implementation of pre‐exposure prophylaxis in Kenya—2017	South Africa: NDoH PrEP implementation pack	Zambia—Implementation framework and guidance for pre‐exposure prophylaxis of HIV infection 2018	Zimbabwe—Implementation plan for HIV pre‐exposure prophylaxis in Zimbabwe 2018–2020	Family planning CIP template	GHCC principles	Consolidated implementation domains/content areas
Planning, leadership and governance^*^ Leadership and governance to increase ownership and coordination. Adaptation and dissemination of guidelines and policies, capacity building and community engagements.	Clinical guideline development	Leadership and governance Sub‐committee National taskforce	National coordination and advocacy for an enabling policy environment	Policy and advocacy to secure resources for plan development stewardship and governance	Start‐up period versus implementation or both	National coordination, policy and planning
		Prioritizing and implementing PrEP services ‐ Populations with high HIV incidence/prevalence by geography, population groups and risk behaviours	Target settings		Target population, coverage, time period	Target setting
Communications, advocacy and community engagement	Communication and community‐based strategies	Build awareness/create demand Mobilize communities	Awareness raising, PrEP promotion	Demand, communication and outreach		Communication/awareness raising/demand creation
Service delivery operations^*^ ‐delivered using community‐based and facility‐based delivery models, ‐prevention centres, pharmacies, stand‐alone DICEs, special clinics MCH/FP/ANCs, youth‐friendly centres, comprehensive care centres, outpatient departments	Quality of care Effectiveness and efficiency Integration across various entry points	Programming PrEP services Service delivery minimum standards where PrEP demand can be generated Integrated in existing services to reach populations Facilities with relevant services, (HIV and STI testing, ART, YFS, MNCH, VMMC, OPD, family planning), facility readiness	Public and private‐sector facility mapping Provision of PrEP	Service delivery	Delivery mechanism	Service delivery approaches
Adherences support	Quality of care: counselling, stigma reduction and adherence					Counselling and adherence support
Human resources In‐service training/pre‐service education Trainer of trainers (TOT)	Human resources	Human resources ‐ integrated HIV care training	Provider sensitization and training	Salary/labour cost		Human resources
PrEP (TDF/FTC)^*^	PrEP (TDF/FTC)	PrEP (TDF/FTC)		Commodities	Intervention	PrEP intervention (commodities)
Laboratory (baseline tests and monitoring) forecasting and quantifying Monitoring for supply security Warehousing and distribution		Laboratory services	Laboratory monitoring services			Laboratory monitoring services and other commodities
Commodity management procedures^*^ (ordering/handling and reporting) Commodity security Logistic management information systems (LMIS): forecasting and quantifying Monitoring for supply security Warehousing and distribution	Functioning supply chain, including drugs and commodities	Procurement and supply chain	Maintain a consistent supply of PrEP medicines	Commodity security		Supply chain management and logistics
Monitoring and evaluation systems (documentation and reporting)		Developing system triggers for people who cannot adhere			Supporting change	Health information systems
Monitoring and evaluation^*^ Quality improvement Facilitate and inform scale up Improving PrEP programme efficiency Continuous quality control and improvement (CQI)	Monitoring and evaluation	Monitoring and evaluation Integrate PrEP monitoring within existing reporting services Assess adherence, retention and linkages Consider risk‐based reasons for stopping	Integrated monitoring and evaluation system for PrEP	Monitoring and coordination		Monitoring and evaluation
Research and impact evaluation			Conduct research and evaluation		Research and supporting change	Implementation science and operations research
Financing and resource mobilization^*^	Costing and financing the PrEP and T&T policy ‐ establish the cost of implementation of these plans at national and provincial levels		Mobilize and track resources	Financing		Budgeting, costing and financing
				Capital	Overhead costs	Indirect/overhead
					Above‐service delivery	Above‐site activities

*Note*: Asterisks (^*^) indicate that the cost was estimated or budgeted within the National plan. Abbreviations: ANCs, Antenatal care; ART, antiretroviral therapy; CIP, costed implementation plans; DICEs, drop‐in centres; FP, family planning; GHCC, Global Health Cost Consortium; MCH, maternal and child health; MNCH, maternal neonatal and child health; NDoH, National Department of Health; OPD, outpatient departments; PrEP, pre‐exposure prophylaxis; STI, sexually transmitted infections; TDF/FTC, tenofovir disoproxil and emtricitabine; VMMC, voluntary medical male circumcision; YFS, youth‐friendly services.

Six of the 15 implementation domains were in five (plans and template): national coordination, policy, and planning; awareness raising and demand creation; service delivery approaches; human resources; supply chain and logistics; and monitoring and evaluation (Table 1). The other domains were included in two or three plans.

### 3.3 Study characteristics

**Figure 1:**
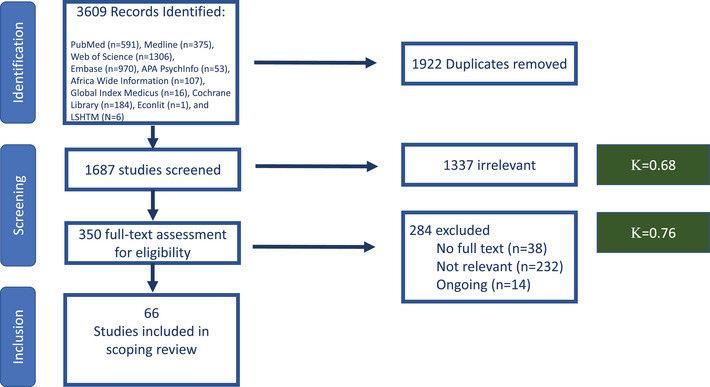
PRISMA diagram of identified, screened and included studies.

Searches of electronic databases for oral PrEP and LAP costing and cost‐effectiveness literature yielded 3609 publications; of which, 1922 were duplicates, 1687 underwent title‐abstract screening, 1337 were excluded and 350 underwent full‐text review. We excluded ongoing studies (*n* = 14) and studies lacking full‐text availability (*n* = 38) or relevancy (*n* = 232), such as only high‐income country settings, not reporting cost data, or reporting only qualitative findings. Ultimately, 66 studies were included (Figure 1). Reviewers exhibited moderate to good agreement during title‐abstract screening (*K* = 0.68) and better agreement in full‐text review (*K* = 0.75).

### 3.4 Populations

The 66 studies (Table 2) represented the following specific population groups: adolescent girls and young women (AGYW) (29%); men who have sex with men (MSM) (27%); sex workers (27%, majority female sex workers except for one reported male sex worker); general population (26%); women of any age (18%); men of any age (9%); and sero‐different couples (SDCs) (17%). The populations least represented (<7%) were adolescent boys and young men (ABYM), people who inject drugs, trans women, pregnant and breastfeeding women, and prisoners. Additional study details of each population group disaggregated cost and economic data are shown in S4A, and for all populations regardless of cost and economic data disaggregation in S4B.

**Table 2:** Characteristics of included studies (N=66).

Author	Year of publication	Countries	Priority population represented	Priority population costed	Primary costing	PrEP methods	Long‐acting PrEP	Aims/objectives
Ying	2015	Uganda	Sero‐different couples (SDC)	SDC	Yes	Daily oral PrEP	No	Estimate the additional operational costs of PrEP delivery in an open‐label, prospective study and project long‐term health and economic outcomes and estimate cost‐effectiveness of PrEP implementation
Eakle	2017	South Africa	Sex worker	Sex worker	Yes	Daily oral PrEP	No	Support integration of oral PrEP, as part of a combination prevention approach, and early antiretroviral therapy (ART) into existing HIV services in two urban settings, with specific aims to assess uptake, retention and adherence among female sex workers and to estimate the cost of this strategy
Suraratdecha	2018	Thailand	Men who have sex with men (MSM)	MSM	Yes	Daily oral PrEP	No	Assess the cost of providing oral PrEP to MSM and estimate the epidemiological impact and cost‐effectiveness of oral PrEP for this target group
Wong	2018	China (Hong Kong)	MSM	MSM	Yes	Daily oral PrEP	No	Examine the impact of PrEP in a setting with low HIV incidence with a low proportion of high‐risk MSM in Asia, through developed an epidemic model and conducted cost‐effectiveness analysis using empirical multicentre clinical and HIV sequence data from MSM living with HIV in Hong Kong, in conjunction with behavioural data of local MSM
Irungu	2019	Kenya	SDC	SDC	Yes	Daily oral PrEP	No	Estimate the cost of delivering antiretroviral‐based HIV prevention to HIV SDC in public health facilities in Kenya and the incremental cost of providing PrEP as a component of this strategy
Roberts	2019	Kenya	Adolescent girls and young women (AGYW)	AGYW	Yes	Daily oral PrEP	No	Estimate the incremental cost of integrating PrEP delivery into routine MCH and FP services and explore the cost implications of service delivery modifications, such as timing of creatinine monitoring and prioritized delivery to women identified as having high risk for HIV acquisition
Hughes	2020	Zimbabwe	SDC	SDC	Yes	Daily oral PrEP	No	Estimate the resources required to deliver various safer conception strategies and calculate the incremental cost per couple for “real world” scenarios for the delivery of the safer strategies in the public sector
Peebles	2021	Kenya	SDC	Other	Yes	Daily oral PrEP	No	Estimate the incremental cost of public‐sector HIV‐1 care clinic‐based provision of PrEP in Kenya
Hendrickson	2021	Zambia	AGYW, general population, MSM, sex worker	AGYW, general population, other	Yes	Daily oral PrEP	No	Present the results of a costing study of PrEP implementation in Zambia, aiming to provide cost estimates of PrEP provision disaggregated by programme type and showcase costs per PrEP‐month of effective use, using aggregate PrEP persistence data, and compare that to costs for perfect use
Wanga	2021	Kenya	AGYW	AGYW	Yes	Daily oral PrEP	No	Evaluate the cost of delivering daily oral PrEP to AGYW in two family planning clinics in Kisumu and estimate the total annual cost and average cost per client‐month of PrEP dispensed as implemented in the study setting and as would be incurred by the Kenyan Ministry of Health if it were to implement PrEP delivery to the same population in the same facilities
Mudimu	2022	South Africa	AGYW	AGYW	Yes	Daily oral PrEP	No	Evaluate the cost of PrEP provision with effective use counselling offered to AGYW through community‐based HIV testing platforms
Mangenah	2022	Zimbabwe	AGYW, men, women	AGYW, men, women	Yes	Daily oral PrEP	No	Provide input into cost‐effectiveness modelling and data for assessing resource needs associated with scaling up PrEP delivery
Okal	2022	Kenya	AGYW	AGYW	Yes	Daily oral PrEP	No	Compare unit costs of providing DREAMS interventions to AGYW across two sites, an urban (Nyalenda A Ward) and peri‐urban (Kolwa East Ward) setting, in Kisumu County, Kenya
Smith	2016	South Africa	Sex worker, ABYM, AGYW, men, women, adolescents	General population	No	bnAbs, PrEP ring, daily oral PrEP, injectable PrEP	Yes	Construct a strategic approach to HIV prevention using limited resources to achieve the greatest possible prevention impact through the use of interventions available today and in the coming years
Stover	2016	Asia and Pacific, East and Southern Africa, Eastern Europe and Central Asia, Latin America, Middle East and North Africa, West and Central Africa, West and Central Europe and North America	AGYW, general population, MSM, people who inject drugs (PWID), prisoners, SDC, sex worker, trans women (TGW)	Other	No	Other PrEP (PrEP includes oral pills, vaginal gel, vaginal ring and injectable forms)	Yes	Describes the analysis that produced the 2020 and 2030 Fast‐Track targets and the estimated resources needed to achieve them in low‐ and middle‐income countries
Walensky	2016	South Africa	Women	Women	No	Daily oral PrEP, injectable PrEP	Yes	Anticipate the development of newer PrEP formulations, investigate the effectiveness thresholds that would justify the additional cost over existing PrEP alternatives in a population of high‐risk young women in South Africa, and identify the key drivers and uncertainties behind that assessment
Glaubius	2016	South Africa	General population	General population	No	Injectable PrEP	Yes	Analyse scenarios of RPV PrEP scale‐up for combination HIV prevention in comparison with a reference scenario without PrEP
Quaife	2018	South Africa	AGYW, sex worker, women	AGYW, sex worker, women	No	Daily oral PrEP, PrEP ring, other PrEP (includes multiple combinations of multi‐purpose oral, vaginal ring, injectable, gels and diaphragm technologies)	Yes	Examine the cost‐effectiveness of the incremental benefits and health system costs of single‐ and multi‐purpose prevention products, compared to current practice of condom use and male circumcision prevalence and model cost‐effectiveness across three female groups: younger women (aged 16–24), older women (aged 25–49) and female sex workers
van Vliet	2019	South Africa	Women	Women	No	Injectable PrEP	Yes	Model how many HIV infections could be averted if injectable contraceptive users started using long‐acting PrEP and determine the cost at which long‐acting PrEP drugs would be cost‐effective
Glaubius	2019	South Africa	Women	Women	No	PrEP ring	Yes	Evaluated the potential epidemiological impact and cost‐effectiveness of dapivirine vaginal ring PrEP among 22‐ to 45‐year‐old women in KwaZulu‐Natal, South Africa
Reidy	2019	Kenya, South Africa, Uganda, Zimbabwe	AGYW, general population, sex worker	AGYW, general population, sex worker	No	Daily oral PrEP, PrEP ring	Yes	Explore the impact and cost‐effectiveness of the PrEP ring in different implementation scenarios alongside scale‐up of other HIV prevention interventions
Vogelzang	2020	South Africa	Adolescent boys and young men (ABYM), men	ABYM, men	No	Daily oral PrEP, injectable PrEP, other PrEP (oral PrEP + injectable PrEP)	Yes	Estimate the incremental cost‐effectiveness of providing oral PrEP, injectable PrEP or a combination of both to heterosexual South African men to assess whether providing PrEP would efficiently use resources
Adeoti	2021	Nigeria	General population	General population	No	Other PrEP (PrEP as a concept)	Yes	Evaluate the impact of PrEP/PEP using a novel artificial intelligence technology, assessing the impact on HIV burden (incidence) and service utilization in a Nigerian HIV treatment centre
Pretorius	2010	South Africa	AGYW	AGYW	No	Daily oral PrEP	No	Evaluate PrEP alongside ART and condom‐use interventions by developing an age‐structured model, which is contextualized to the South African epidemic, paying attention to the distribution of relative infection risks between age categories
Hallett	2011	South Africa	SDC	SDC	No	Daily oral PrEP	No	Examine impact and cost‐effectiveness of different TasP and oral PrEP strategies
Gomez	2012	Peru	MSM, sex worker, TGW	MSM, sex worker, TGW	No	Daily oral PrEP	No	Investigate the impact of a feasible intervention, determine the most efficient strategies for rollout and examine the impact of coverage, adherence and prioritization on both health benefits and costs to the health system
Long	2013	South Africa	Men, women	Men, women	No	Daily oral PrEP	No	Assess the impact of simultaneously scaling up multiple biomedical HIV prevention programmes and calculate the benefits of reduced secondary transmission among partners of programme recipients
Cremin	2013	South Africa	ABYM, AGYW	Adolescents and young adults	No	Daily oral PrEP	No	Estimate the potential impact and cost‐effectiveness of antiretroviral‐based HIV prevention strategies
Nichols	2013	Zambia	Other	General population	No	Daily oral PrEP	No	Explore the possibilities of daily oral PrEP optimization using realistic data collected in the rural HIV clinic at the Macha Mission Hospital in Zambia and evaluate the risk for resistance development
Verguet	2013	42 Sub‐Saharan countries	General population	General population	No	Daily oral PrEP	No	Study the potential impact and incremental cost‐effectiveness of providing PrEP over a 5‐year period (2013–2017) to a general adult population in sub‐Saharan Africa to provide insight into where and why a PrEP intervention could be best put to use for HIV prevention
Stover	2014	25 Low‐ and middle‐income countries	Adolescents and young adults, general population, MSM, other, SDC, sex worker	Adolescents and young adults, general population, MSM, other, SDC, sex worker	No	Daily oral PrEP, HIV vaccine	No	Examine the impact of achieving high coverage of all existing HIV prevention interventions and three new approaches on the HIV epidemic in all low‐ and middle‐income countries
Nichols	2014	Zambia	Other	General population	No	Daily oral PrEP	No	Compare the cost‐effectiveness and economic affordability of antiretroviral‐based prevention strategies in rural Macha, Zambia
Anderson	2014	Kenya	Sex worker, MSM, men, women	General population	No	Daily oral PrEP	No	Examine how a fixed amount of resources for HIV prevention can be used to generate reductions in the rate of new HIV infections using two forms of resource allocation: (1) the rollout of particular interventions is uniform across the country; and (2) interventions can be focused on geographic or key affected populations that contribute to HIV strongholds
Alistar	2014	South Africa	General population	Other	No	Daily oral PrEP	No	Study the population health outcomes and cost‐effectiveness of implementing expanded ART coverage and oral PrEP in a setting with a heavy HIV burden
Alistar	2014	Ukraine	PWID	PWID	No	Daily oral PrEP	No	Evaluate the cost‐effectiveness of PrEP for PWID alone or as part of a portfolio of interventions including methadone maintenance treatment for PWID and antiretroviral treatment for all individuals living with HIV and project the evolution of the epidemic under various combinations of strategies for HIV control: oral PrEP programmes for uninfected IDUs, MMT programmes for PWID and scale‐up of ART programmes for eligible people living with HIV (including PWID and non‐PWID)
Cremin	2015	Mozambique	Women	Women	No	Daily oral PrEP	No	Estimate the prevention impact and the cost‐effectiveness of providing time‐limited PrEP to partners of migrant miners in Gaza, Mozambique
Jewell	2015	South Africa	SDC	SDC	No	Daily oral PrEP	No	Estimate the cost‐effectiveness of daily oral tenofovir‐based PrEP, with a protective effect against HSV‐2 as well as HIV‐1, among HIV‐1 SDC in South Africa
Cremin	2015	Kenya	General population	General population	No	Daily oral PrEP	No	Investigate the influence of potential interactions between key aspects of a PrEP intervention on projections of epidemiological impact and cost‐effectiveness
Mitchell	2015	Nigeria	SDC	SDC	No	Daily oral PrEP	No	Estimate the impact and cost‐effectiveness of PrEP, TasP and condom promotion for SDC in Nigeria
Price	2016	Sub‐Saharan Africa/Zambia	Pregnant and breastfeeding women	Pregnant and breastfeeding women	No	Daily oral PrEP	No	Develop a decision analytic model to evaluate a strategy of daily oral PrEP during pregnancy and breastfeeding in SSA
Moodley	2016	South Africa	Adolescents and young adults	Adolescents and young adults	No	Daily oral PrEP, HIV vaccine	No	Economically evaluate individual and combination HIV preventive strategies and compare their impact against both the current rollout of ART and a potential scaling‐up of the ART programme
Meyer‐Rath	2017	South Africa	AGYW, sex worker	AGYW, sex worker	No	Daily oral PrEP	No	Identify the optimal mix of HIV services under a constrained budget for the South African HIV Investment Case
Chiu	2017	South Africa	AGYW, sex worker	AGYW, sex worker	No	Daily oral PrEP	No	Describe optimization routines developed for the South African HIV Investment Case and compare its results with those generated using conventional cost‐effectiveness analysis methods to examine the incremental benefit of accounting for interaction effects between interventions and non‐linear effects across scale up
Cremin	2017	Kenya	MSM, sex worker	MSM, sex worker	No	Daily oral PrEP	No	Identify an optimal portfolio of interventions to reduce HIV incidence for a given budget and determine the circumstances in which PrEP could be used in Nairobi, Kenya
Akudibillah	2017	South Africa	General population, sex worker	Other	No	Daily oral PrEP	No	Inform drug‐allocation policy in resource‐limited settings by using a compartmental mathematical model for heterosexual transmission of HIV with treatment targeted by infection status, sexual‐activity level and gender
Alsallaq	2017	Kenya	AGYW	AGYW	No	Daily oral PrEP	No	Compared the impact and costs of HIV prevention strategies focusing on youth (15‐ to 24‐year‐old persons) versus on adults (15+ year‐old persons) in a high‐HIV burden context of a large, generalized epidemic
Anderson	2018	Kenya	Men, MSM, sex worker, women	Men, MSM, sex worker, women	No	Daily oral PrEP	No	Quantify the cost of short‐term funding arrangements on the success of future HIV prevention programmes
Li	2018	China	MSM	MSM	No	Daily oral PrEP	No	Assess the benefits of full implementation of current policies and the timely introduction of novel policies and makes recommendations for future HIV policy responses in China
Luz	2018	Brazil	MSM	MSM	No	Daily oral PrEP	No	Analyse daily tenofovir/emtricitabine PrEP use in MSM and TGW at high risk of HIV in Brazil using the best available epidemiological, clinical and economic data
Stopard	2019	South Africa, Tanzania	General population	General population	No	Daily oral PrEP	No	Investigate how “real‐world” constraints on the allocative and technical efficiency of HIV prevention programmes affect resource allocation and number of infections averted
Zhang	2019	China	MSM	MSM	No	Daily oral PrEP	No	Evaluates the epidemiological impact and cost‐effectiveness of implementing PrEP in Chinese MSM over the next two decades
Bórquez	2019	Peru	TGW	TGW	No	Daily oral PrEP	No	Investigate the status of HIV prevention and delivery of care in Peru in terms of infrastructure, staff capacity, budget allocation, activities, organization and outputs; explore perceptions of HIV risk and knowledge of, attitudes towards and intention to use diverse prevention methods among members of the MSM and transgender women communities, as well as adoption by health professionals and decision‐makers; and estimate the impact and cost‐effectiveness of the various interventions to identify cost‐effective and feasible combinations in the Peruvian setting
Selinger	2019	South Africa	General population	General population	No	Daily oral PrEP, event‐driven PrEP	No	Inform ongoing vaccine access planning elements, including priority populations for whom the pox‐protein HIV vaccine would be expected to have the greatest and/or most efficient public health impact
Hu	2019	China	MSM	MSM	No	Daily oral PrEP	No	Evaluate reductions in HIV transmission that may be achieved through early initiation of ART plus partners’ PrEP
Grant	2020	Kenya, South Africa, Zimbabwe	AGYW, sex worker, women	AGYW, sex worker, women	No	Daily oral PrEP	No	Highlight key considerations to feed into policymaking, as countries consider scaling up PrEP across a more broadly defined group of women at risk in sub‐Saharan Africa, and present decision‐makers with a range of important considerations, including PrEP cost‐effectiveness, cost and estimated number of HIV acquisitions averted on PrEP for different groups of women at population level
Kazemian	2020	India	MSM, PWID	MSM, PWID	No	Daily oral PrEP, event‐driven PrEP	No	Examine the cost‐effectiveness of both PrEP and HIV testing strategies for MSM and PWID in India
Pretorius	2020	Eswatini, Ethiopia, Haiti, Kenya, Lesotho, Malawi, Mozambique, Namibia, Nigeria, Tanzania, Uganda, Zambia, Zimbabwe	AGYW, SDC, sex worker	Other	No	Daily oral PrEP	No	Estimated the impact, cost and cost‐effectiveness of scaling up oral PrEP in 13 countries
Jamieson	2020	South Africa	ABYM, AGYW, MSM, pregnant and breastfeeding women, sex worker	ABYM, AGYW, MSM, pregnant and breastfeeding women, sex worker	No	Daily oral PrEP	No	Analyse the epidemiological impact of PrEP provision to adolescents, young adults, pregnant women, female sex workers, and MSM and estimate the cost and cost‐effectiveness of PrEP
Kazemian	2020	India	MSM	MSM	No	Daily oral PrEP	No	Develop, validate and demonstrate a novel, practical method to estimate the community benefit of HIV interventions that help prevent transmission of HIV without a dynamic transmission model
Wu	2021	China	SDC	SDC	No	Daily oral PrEP	No	Evaluate health economics of antiretroviral‐based strategies for HIV SDC in China
Phillips	2021	South Africa	AGYW, general population, sex worker	General population, other	No	Daily oral PrEP	No	Predict the impact and cost‐effectiveness of PrEP with use concentrated in periods of condomless sex, accounting for effects on drug resistance
Ten Brink	2022	Cambodia, China, India, Indonesia, Myanmar, Nepal, Thailand, Vietnam	MSM	MSM	No	Daily oral PrEP	No	Estimate the impact and cost‐effectiveness of daily versus event‐driven dosing of PrEP for eight Asian countries and compare branded with generic PrEP in China
Kripke	2022	Lesotho, Mozambique, Uganda	General population	General population	No	Daily oral PrEP	No	Examine the role and cost‐effectiveness of HIV prevention in the context of “universal test and treat” in three sub‐Saharan countries with generalized HIV epidemics
Jin	2022	China	MSM	MSM	No	Daily oral PrEP	No	Evaluate the HIV epidemic under several PrEP coverages with or without expanded ART and calculate the cost‐effectiveness of various PrEP scenarios
Phillips	2022	Sub‐Saharan Africa/South Africa	General population	General population	No	Daily oral PrEP	No	Explore the conditions under which widely accessible PrEP could be cost‐effective in sub‐Saharan Africa, assuming a concentration of PrEP use during periods of risk with high adherence to daily pill‐taking
Ghayoori	2022	Rwanda	Women	Women	No	Daily oral PrEP	No	Examine transmission of HIV among female sex workers, general population, sex clients and MSM to inform scaling up PrEP beyond the highest risk population is considered via an analysis of cost‐effectiveness

*Note*: Adeoti (2021) did not refer to the route of administration or PrEP modality in their paper, only “pre‐exposure prophylaxis.” Given when the article was published, we assumed this conceptual mention of PrEP included long‐acting methods, along with daily oral PrEP.

Abbreviations: ABYM, adolescent boys and young men; AGYW, adolescent girls and young women; ART, antiretroviral therapy; DREAMS, Determined, Resilient, Empowered, AIDS‐free, Mentored, Safe; MMT, methadone maintenance treatment; MSM, men who have sex with men; PrEP, pre‐exposure prophylaxis; PWID, people who inject drugs; TasP, treatment‐as‐prevention; TGW, trans women.

### 3.5 Geography

**Figure 2:**
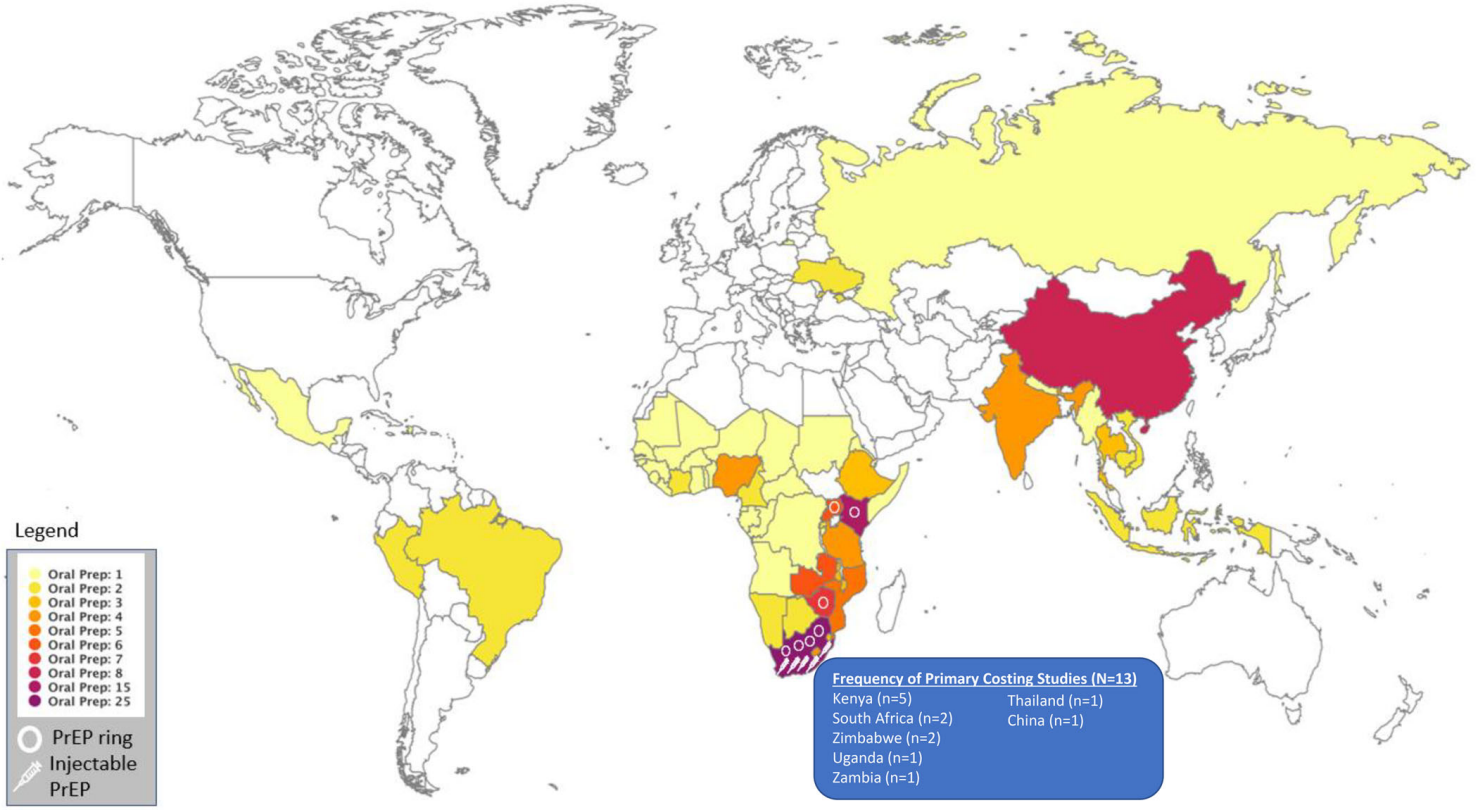
Distribution of countries and PrEP methods represented in sample of costing studies (*N* = 66).

The 66 studies, representing 69 countries (Figure 2), and one study representing all of SSA without sufficient details to make a country assignment were included. Most studies represented a few countries: the top six countries were South Africa (44%, *n* = 29), Kenya (24%, *n* = 16), China (14%, *n* = 9), Zimbabwe (12%, *n* = 8) and Uganda & Zambia (11%, *n* = 7). Eleven studies were multicounty. Studies represented the following UNAIDS geographic regions: Southern and Eastern Africa (*n* = 47), West and Central Africa (*n* = 4), Asia and the Pacific (*n* = 12), Eastern Europe and Central Asia (*n* = 2), Latin America and the Caribbean (*n* = 5), and the Middle East and North Africa (*n* = 1). Additional details by region and country are shown in S5A for regions or countries with disaggregated cost and economic data and S5B for all countries cited regardless of disaggregation.

### 3.6 Long‐acting PrEP studies

**Figure 3:**
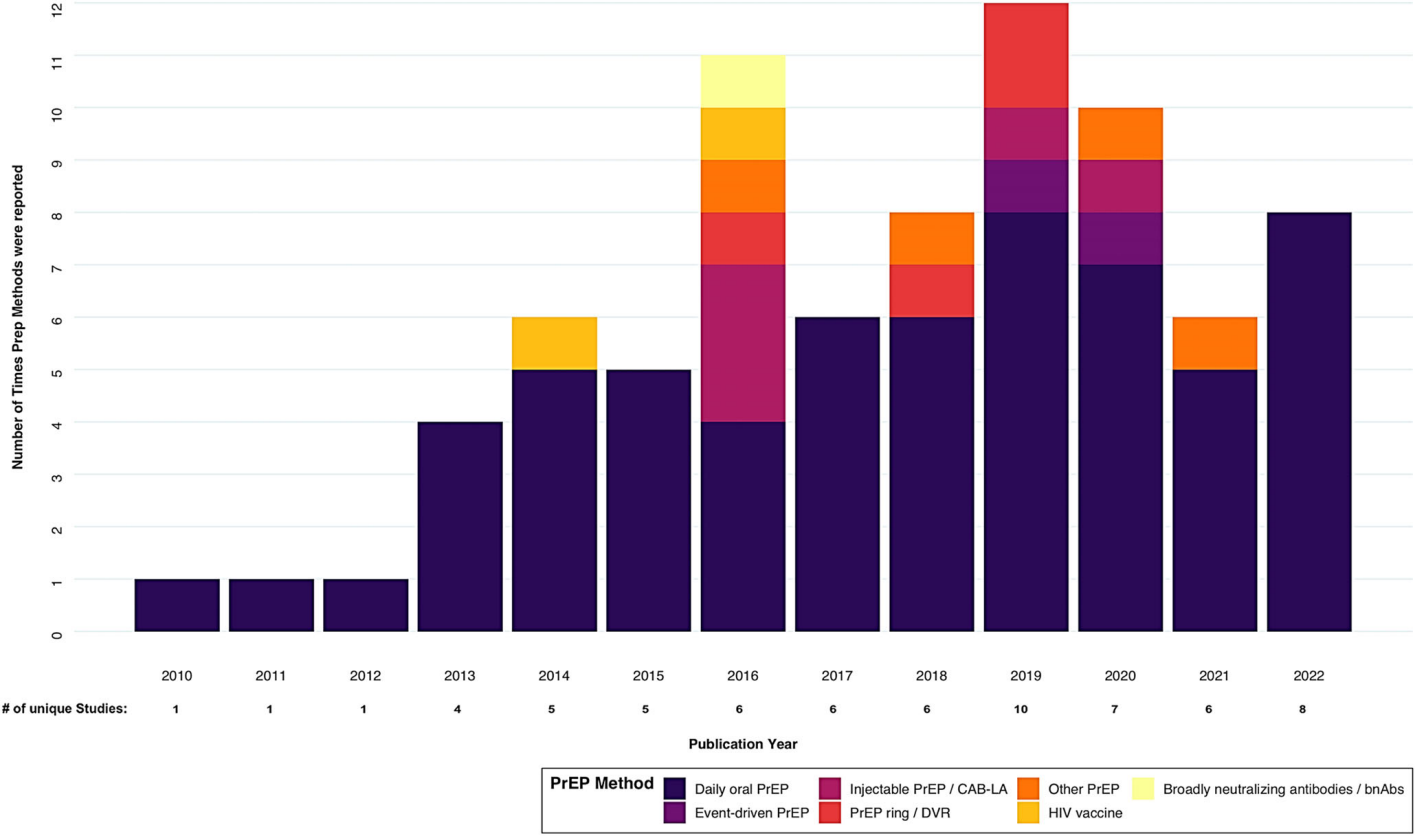
Frequency of reported PrEP methods, by number of unique costing studies and publication year.

Figure 3 shows the frequency of studies by year and the PrEP method reported. Approximately 15% (*n* = 10) were specifically LAP studies (Table 2), with two reporting injectable PrEP, one reporting PrEP ring, and seven reporting multiple forms or combinations of LAP. In total, five reported injectable PrEP, four reported on the PrEP ring, and four reported combinations of methods, such as dual HIV and pregnancy prevention or without enough specificity to define the type of LAP. The LAP studies focused on general population women (5), AGYW (4) and female sex workers (3) primarily (Table 2). The 14 country results from 10 LAP studies were mainly conducted in South Africa (*n* = 8), with results from Nigeria, Zimbabwe, Kenya and Uganda. One study reported LAP data for all global regions (Figure 2).

### 3.7 Description of implementation domains reported in primary cost evaluations

Table 3 displays the distribution of implementation domains among the included studies and Table 4 for LAP studies specifically. Since purpose, scope, and methods can differ by study type, we stratified these findings to discern primary cost studies from secondary or modelled evaluations. Given their direct implications for implementation, we focus our findings on the primary costing studies below.

**Table 3:** Frequency of key PrEP implementation domains for all studies, by biomedical HIV prevention method and costing approach

	Total			Daily oral PrEP		Event‐driven PrEP		Injectable PrEP		PrEP ring		Other PrEP			
Costed implementation domains	All *N* = 66)	Primary costing (*n* = 13)	Secondary costing or modelling (*n* = 53)	Primary costing (*n* = 13)	Secondary costing or modelling (*n* = 48)	Primary costing (*n* = 0)	Secondary costing or modelling (*n* = 2)	Primary costing (*n* = 0)	Secondary costing or modelling (*n* = 5)	Primary costing (*n* = 0)	Secondary costing or modelling (*n* = 4)	Primary costing (*n* = 0)	Secondary costing or modelling (*n* = 4)	Primary costing references	Secondary costing or modelling references
National coordination, policy and planning	3	2	1	2	1				1		1			47, 52	84
Target setting															
Human resources	30	11	19	11	16				2		2		2	42, 44, 51, 45, 50, 49, 43, 47, 52, 46, 53	96, 95, 132, 98, 84, 89, 133, 135, 114, 125, 122, 88, 109, 87, 110, 127, 128, 93, 111
Communication/awareness raising/demand creation	21	9	12	9	11				2		2		1	42, 44, 45, 50, 49, 43, 47, 46, 53	96, 84, 89, 133, 135, 113, 114, 122, 88, 109, 127, 128
Counselling and adherence support	25	9	16	9	15				1		1		1	42, 44, 45, 50, 49, 43, 48, 47, 46	97, 96, 89, 134, 113, 88, 109, 110, 123, 124, 94, 127, 119, 128, 111, 105
PrEP intervention (commodities)	38	12	26	12	32		1		3		4		4	42, 44, 45, 50, 49, 43, 83, 48, 47, 52, 46, 53	96, 95, 132, 98, 84, 108, 134, 135, 113, 114, 86, 125, 122, 88, 109, 123, 124, 116, 127, 91, 128
Supply chain management and logistics	6	3	3	3	2									47, 52, 53	84, 135, 125
Laboratory and other commodities	28	10	18	10	17		1		2		2		1	42, 44, 51, 45, 50, 49, 43, 48, 47, 46	96, 95, 132, 89, 108, 134, 135, 113, 114, 86, 122, 88, 123, 124, 116, 127, 91, 128
Health information systems															
Service delivery approaches	13	5	8	5	6				2		1		1	42, 44, 45, 83, 52	89, 125, 88, 87, 110, 128, 111, 105
Monitoring and evaluation	11	7	4	7	3				2				1	42, 51, 45, 49, 83, 47, 53	96, 89, 92, 128
Implementation science and operations research															
Above‐site activities	5	3	2	3	2									47, 52, 46	113, 122
Indirect/overhead	20	11	9	11	6				2		1		2	42, 44, 51, 45, 50, 49, 43, 47, 52, 46, 53	98, 89, 114, 88, 87, 124, 127, 128, 93
Other costs	14	7	7	7	6								1	42, 51, 45, 50, 43, 47, 53	98, 135, 99, 116, 127, 93, 129

*Note*: The one PrEP‐bnAbs secondary costing or modelling study (Citation 84) costed policy & planning, awareness raising & demand generation, PrEP commodities and supply chain logistics & management. Of the two PrEP‐HIV vaccine secondary costing or modelling studies, one (Citation 111) costed human resources, counselling and PrEP integration into non‐HIV services.

Among primary costing studies reporting integration data (*N* = 5), studies costed the integration of PrEP into sexual and reproductive health services (*n* = 4), family planning services (*n* = 3), and maternal and child health services (*n* = 1). For secondary costing or modelling studies (*N* = 6), sexual and reproductive health services was the most common (*n* = 4), followed by family planning (*n* = 1), antenatal care (*n* = 1), and HIV treatment and methadone maintenance treatment (*n* = 1).

Abbreviation: PrEP, pre‐exposure prophylaxis.

**Table 4:** Frequency of key PrEP implementation domains for all LAP studies (*N* = 10)

Costed implementation domains	Total *N* = 10	Injectable PrEP *n* = 5	PrEP ring *n* = 4	Other PrEP *n* = 4	References
National coordination, policy and planning	1	1	1		84
Target setting					
Human resources	5	2	2	1	84, 87, 88, 89, 93
Communication/awareness raising/demand creation	3	2	2	1	84, 88, 89
Counselling and adherence support	2	1	1	1	88, 89
PrEP intervention (commodities)	9	3	3	4	84, 85, 86, 92, 88, 87, 90, 91, 93
Supply chain management and logistics	1				
Laboratory and other commodities	4	2	2	1	86, 88, 89, 91
Health information systems					
Service delivery approaches	3	2	1	1	87, 88, 89
Monitoring and evaluation	2	2		1	89, 92
Implementation science and operations research					
Above‐site activities					
Indirect/overhead	4	2	1	2	87, 88, 89, 93
Other costs				1	93

Abbreviations: LAP, long‐acting pre‐exposure prophylaxis; PrEP, pre‐exposure prophylaxis.

Among the 13 primary cost studies, the most reported implementation domains were: PrEP‐intervention‐commodities (*n* = 12), laboratory and other commodities (*n* = 10), human resources (*n* = 11), indirect/overhead costs (*n* = 11), communication/awareness raising/demand creation (*n* = 9), counselling and adherence support (*n* = 9), and monitoring and evaluation (*n* = 7). A few studies included the cost of integration into non‐HIV services (*n* = 5), above‐site activities (*n* = 3), supply chain management and logistics (*n* = 3), or national coordination policy and planning (*n* = 2). No primary cost study included target setting, health information systems, or research. None of the primary costed studies included LAP. Of the 13 primary cost studies, 53.8% (*n* = 7) were conducted in government or public facilities (Table 5). Most primary studies estimated average costs (*n* = 9) and incremental costs (*n* = 7). Three studies also modelled cost‐effectiveness. Most of the costing studies utilized the health system perspective (*n* = 11) and applied a discount rate (*n* = 11). Two studies did not report conflict of interest statements. Similar details for modelled studies are reported in S6.

**Table 5:** Economic analysis features and analytic approaches of primary costing studies (N = 13).

	Total (*N* = 13)	Daily oral PrEP (*n* = 13)	Event‐driven PrEP (*n* = 0)	Injectable PrEP (*n* = 0)	PrEP ring (*n* = 0)	Other PrEP (*n* = 0)	References
**Facility type**							
NGO & NGO facility	5	5					42, 44, 51, 45, 52
Private‐for‐profit & private non‐profit & private facility	2	2					52, 53
Government & public facility	7	7					44, 49, 43, 48, 47, 52, 53
**Type of economic analysis**							
Cost‐effectiveness	8	8					42, 44, 45, 50, 43, 83, 48, 47
Cost‐benefit							
Cost‐utility	3	3					44, 83, 48
Average costing	9	9					51, 45, 50, 49, 43, 47, 52, 46, 53
Incremental costing	7	7					42, 44, 45, 43, 83, 48, 47
**Costing approach**							
Guideline/normative	3	3					51, 50, 83
Real world/actual	5	5					42, 44, 49, 48, 47
Both	5	5					45, 43, 52, 46, 53
**Economic v. financial costing**							
Economic costing	9	9					44, 51, 45, 49, 43, 47, 52, 46, 53
Financial costing	4	4					42, 50, 83, 48
**Perspective**							
Health system	11	11					42, 44, 51, 45, 50, 49, 43, 48, 52, 46, 53
Societal							
Both	1	1					47
Not reported	1	1					83
**Discount rate reported**	11	11					42, 44, 51, 50, 49, 43, 83, 48, 47, 52, 53
**Conflict of interest**							
Yes	1	1					46
No	10	10					42, 44, 51, 45, 50, 49, 43, 83, 52, 53
Not reported	2	2					48, 47
**Approach**							
Observed	9	9					42, 44, 51, 49, 43, 47, 52, 46, 53
Observed + modelled	4	4					45, 50, 83, 48

Abbreviations: NGO, non‐governmental organization; PrEP, pre‐exposure prophylaxis.

### 3.8 Implementation assumptions in primary cost evaluations

All primary cost studies reported costs in USD; 11 studies included overhead costs. All studies reporting discount rates (*N* = 11) used 3%. Considerable heterogeneity persisted in the activities described in the primary cost studies, the costs estimated, and the units defined in all other implementation domains. For instance, within the commonly reported PrEP commodities domain, average drug costs were described as cost per implementation scenario [42, 43], costs across all sites and per site [44], cost per couple [43, 45], cost per client [46, 47, 48], costs per bottle [43, 49, 50], per person‐year [43, 50], and per month [45, 47]. The recurrent drug costs cited ranged from $24.43 to $382.00. PrEP commodities were only included if the input costs of the drug were explicitly reported, could be disaggregated or were cited as being included in the unit cost. The human resources domain included clinicians, social workers, and counsellors staff time based on time and motion studies [42]. Other studies included the site doctor, pharmacist, peer educators [44], and administrators [51]. Merely three studies described start‐up training [45, 46, 49]. For the more infrequently costed domains, policy and planning costs were estimated for stakeholder coordination required to start up and microplanning [47, 52], and supply chain costs were estimated for central storage and distribution fees and a one‐time PrEP importation fee [47, 52, 53].

### 3.9 Primary costing study outcomes

Table 7 summarizes average and incremental cost outcomes reported across the 13 primary cost studies and Tables 8 and 9 detail cost and cost‐effectiveness outcomes of studies by scenarios costed. The 13 studies reported 11 unique outcome indicators. Cost per person/client‐month (*n* = 6) was the most frequently reported outcome, followed by annual costs (*n* = 4), cost per visit (*n* = 4), and cost per person/client‐year (*n* = 3). Three primary costing studies also reported the results of cost‐effectiveness analyses: one study reported an incremental cost‐effectiveness ratio (ICER) without clearly defining the time horizon; one reported an ICER (cost per acquisition averted) over a 10‐year horizon; and the third reported the incremental cost‐effectiveness per quality‐adjusted life year (QALY) gained in a 5‐year time horizon. No single cost or cost‐effectiveness outcome was measured by all studies utilizing the same analytic approach. The highest frequency of studies reported on oral PrEP among AGYW, followed by SDC.

### 3.10 Implementation domains in secondary data or modelled studies

A lower percentage of modelled than primary costing studies included the costs of any implementation. Among the 53 modelled studies (Table 6), the cost of PrEP commodities was the most frequently included (40%, *n* = 21), followed by human resources (36%, *n* = 19). Table 6 summarizes all modelled studies. As shown in S6, most modelled studies were cost‐effectiveness (*N* = 41), followed by cost‐utility studies (*N* = 15). Evaluations mostly took a guidelines approach (*N* = 32), from the health system perspective (*N* = 42). About 69.8% reported discount rates (*N* = 37) (Tables 6).

**Table 6:** Economic analysis features and analytic approaches of secondary costing or modelling studies (N = 53).

	Total (*N* = 53)	Daily oral PrEP (*n* = 48)	Event‐driven PrEP (*n* = 2)	Injectable PrEP (*n* = 5)	PrEP Ring (*n* = 4)	Other PrEP (*n* = 4)	References
**Facility type**							
NGO & NGO facility	4	4					125, 122, 99, 116
Private‐for‐profit & private non‐profit & private facility	1	1					125
Government & public facility	3	3					95, 113, 122
**Type of economic analysis**							
Cost‐effectiveness	41	40	1	3	1	2	97, 96, 95, 132, 98, 120, 130, 89, 106, 107, 108, 101, 133, 135, 136, 113, 117, 114, 86, 118, 125, 121, 122, 92, 102, 88, 109, 99, 123, 124, 94, 116, 103, 127, 91, 119, 100, 128, 111, 129, 105
Cost‐benefit							
Cost‐utility	15	14	1	2		1	96, 130, 89, 107, 108, 101, 133, 113, 117, 118, 92, 123, 124, 100, 105
Average costing	12	11			2	1	97, 98, 135, 125, 122, 115, 88, 124, 94, 127, 91, 128
Incremental costing	8	7				1	97, 98, 130, 88, 116, 91, 128, 105
**Costing approach**							
Guideline/normative	32	28	2	2	3	2	97, 96, 95, 98, 120, 89, 107, 117, 125, 121, 122, 102, 126, 115, 88, 87, 110, 131, 99, 123, 94, 116, 103, 104, 90, 127, 91, 100, 128, 93, 111, 105
Real world/actual	1	1					124
Both	14	13		3	1	2	132, 130, 84, 106, 108, 101, 133, 112, 85, 113, 114, 86, 118, 92
Not reported	6	6					134, 135, 136, 109, 119, 129
**Economic v. financial costing**							
Economic costing	32	29	1	3	4	2	96, 95, 132, 98, 84, 89, 107, 101, 133, 134, 85, 113, 114, 86, 118, 125, 122, 115, 88, 99, 123, 124, 94, 116, 103, 104, 90, 127, 91, 119, 100, 105
Financial costing	15	14	1	2		1	97, 120, 130, 106, 108, 112, 117, 92, 102, 126, 87, 110, 131, 128, 111
Not reported	6	5				1	135, 136, 121, 109, 93, 129
**Full v. incremental costing**							
Full (Average)	11	11	1	1	2		120, 84, 115, 131, 123, 124, 94, 103, 91, 119, 129
Incremental	5	4			1		134, 136, 104, 90, 127
Both	34	31	1	4	1	3	97, 96, 95, 132, 98, 130, 89, 106, 107, 108, 101, 133, 112, 85, 113, 117, 114, 86, 118, 125, 121, 122, 92, 102, 126, 88, 109, 87, 110, 99, 116, 100, 111, 105
Not reported	3	2				1	135, 128, 93
**Perspective**							
Health system	42	39	1	4	4	3	97, 96, 95, 132, 98, 84, 89, 106, 107, 108, 101, 133, 135, 112, 85, 113, 117, 114, 86, 118, 125, 122, 92, 88, 109, 110, 131, 99, 123, 94, 116, 103, 104, 90, 127, 91, 119, 100, 128, 111, 129, 105
Societal	5	4	1	1			130, 126, 115, 87, 124
Both							
Not reported	6	5				1	120, 134, 136, 121, 102, 93
**Discount rate reported**	37	34	2	5	3	2	97, 95, 132, 98, 130, 84, 89, 106, 107, 108, 101, 133, 134, 135, 86, 118, 121, 122, 92, 102, 126, 88, 109, 87, 110, 131, 99, 123, 124, 116, 104, 90, 119, 100, 111, 129, 105
**Conflict of interest**							
Yes	12	10	1	3			89, 106, 133, 135, 117, 114, 86, 118, 121, 126, 87, 131
No	27	26	1	2	2	3	97, 132, 98, 120, 130, 84, 108, 101, 134, 112, 85, 113, 125, 122, 92, 102, 115, 88, 109, 110, 99, 123, 116, 119, 100, 128, 129
Not reported	14	12			2	1	96, 95, 107, 136, 124, 94, 103, 104, 90, 127, 91, 93, 111, 105
**Approach**							
Observed	8	5	1		1	1	120, 126, 115, 88, 103, 104, 90, 91
Modelled	49	44	1	5	3	4	97, 96, 95, 132, 98, 120, 130, 84, 89, 106, 107, 108, 101, 133, 134, 135, 136, 112, 85, 113, 117, 114, 86, 118, 125, 121, 122, 92, 102, 126, 88, 109, 87, 110, 131, 99, 123, 124, 94, 116, 127, 91, 119, 100, 128, 93, 111, 129, 105

Abbreviations: NGO, non‐governmental organization; PrEP, pre‐exposure prophylaxis.

**Table 7:** Economic analysis features and analytic approaches of secondary costing or modelling studies

Author	Year	Country	PrEP method	Population	Total costs	Annual costs	Total 5‐year	Total 10‐year	Cost per person/client	Cost per person/client‐month	Cost per person/client‐year	Cost per couple	Cost per couple‐year	Cost per visit (initiation, follow‐up, any visit)	Total recurrent cost per PrEP‐client per year	Discounted incremental cost of PrEP strategies over 5‐year	Incremental cost of PrEP per couple	Annual incremental cost
Suraratdecha	2018	Thailand	Daily oral PrEP	MSM		✓	✓			✓								
Wong	2018	China (Hong Kong)	Daily oral PrEP	MSM												✓		
Ying	2015	Uganda	Daily oral PrEP	SDC				✓									✓	
Eakle	2017	South Africa	Daily oral PrEP	Sex worker							✓			✓				
Roberts	2019	Kenya	Daily oral PrEP	AGYW		✓				✓				✓				
Irungu	2019	Kenya	Daily oral PrEP	SDC		✓							✓				✓	✓
Hughes	2020	Zimbabwe	Daily oral PrEP	SDC								✓						
Peebles	2021	Kenya	Daily oral PrEP	Other (see below)	✓				✓	✓								
Wanga	2021	Kenya	Daily oral PrEP	AGYW		✓				✓				✓				
Hendrickson	2021	Zambia	Daily oral PrEP	AGYW/GP/other (FSW & MSM together)						✓	✓				✓			
Okal	2022	Kenya	Daily oral PrEP	AGYW	✓				✓									
Mudimu	2022	South Africa	Daily oral PrEP	AGYW						✓								✓
Mangenah	2022	Zimbabwe	Daily oral PrEP	AGYW/men/women							✓			✓				
Total					2	4	1	1	2	6	3	1	1	4	1	1	2	2

*Note*: When studies listed costs as “Total annual,” we recorded them as Annual costs and only noted Total costs if they were framed exactly as such (i.e. “Total costs”).

Abbreviations: AGYW, adolescent girls and young women; MSM, men who have sex with men; PrEP, pre‐exposure prophylaxis; SDC, sero‐different couples.

**Table 8:** Cost outcomes of primary costing studies

Author	Population(s)	Scenarios	Findings	Sensitivity analysis performed
Suraratdecha	MSM	Cohort study (includes MOPH and project staff), which presented two implementation options: Option 1: one visit for initial PrEP counselling and recruitment, four additional HIV tests, two tests for creatinine, one HBs Ag test, 12 months TDF/FTC combination, six visits for maintenance support (counselling) Option 2: option 1 package plus two times upgraded STIs screening (chlamydia, gonorrhoea, syphilis rapid test, nucleic acid amplification test)	Cohort study total annual costs associated with PrEP initiation and clinic visits Personnel: $1452, Lab supplies: $1406, PrEP drugs: $14,106, Other supplies: $242 Total: $17,206 Option 1: Unit cost of PrEP recommended package (per person per year) Personnel: $24.66, Supplies: $10.36, Tenofovir/Entricitabine (12 bottles: 1 pill/day): $186.33 Total unit cost: $221.34 Total unit cost with 0.7% overhead: $222.89 Total unit cost with 0.7% overhead and 22% demand generation activities: $271.59 Option 2: Unit cost of PrEP recommended package (per person per year) Personnel: $25.63, Supplies: $41.41, Tenofovir/Entricitabine (12 bottles: 1 pill/day): $186.33 Total unit cost: $253.37 Total unit cost with 0.7% overhead: $255.14 Total unit cost with 0.7% overhead and 22% demand generation activities: $310.88	No (Cost‐effectiveness only)
		Providing PrEP to only high‐risk MSM (defined as having engaged in condomless sex with casual or known HIV‐positive partners) versus all MSM, regardless of risk	TOTAL 5‐YEAR PROGRAMME COST: PrEP provided to High‐risk MSM $41.99 (M) PrEP provided to All MSM $147.14 (M)	
Wong	MSM	Basecase with 10%, 30% and 90% coverage of PrEP involving low‐risk and high‐risk MSM (i.e. non‐targeting approach) with low or high adherence usage and high‐risk MSM only (i.e. targeting approach) with low or high adherence usage Plans (apply to both scenarios) Plan A: PrEP priced at the market rate: $7880/year Plan B: PrEP priced at the generic rate: $519/year Plan C: PrEP is free	DISCOUNTED INCREMENTAL COST OF PrEP STRATEGIES OVER 5‐YEAR TIME HORIZON Plan A Respective costs of Non‐targeting 10%; 30%; and 90% are $123,458,936; $370,266,861; and $1,113,780,354 Respective costs of Targeting 10%; 30%; and 90% are $52,571,166; $157,200,505; and $472,011,282 Plan B Respective costs of Non‐targeting 10%; 30%; and 90% are $17,294,670; $51,648,582; and $157,156,635 Respective costs of Targeting 10%; 30%; and 90% are $7,459,389; $21,831,597; and $65,661,580 Plan C Respective costs of Non‐targeting 10%; 30%; and 90% are $9,806,914; $29,176,464; and $89,686,050. Respective costs of Targeting 10%; 30%; and 90% are $4,277,659; $12,284,040; and $37,001,772	Yes
		Test‐and‐Treat included a high rate of diagnosis and treatment initiation (minimum 90% from 2017) with 10%, 30% and 90% coverage of PrEP involving low‐risk and high‐risk MSM (i.e. non‐targeting approach) with low or high adherence usage and high‐risk MSM only (i.e. targeting approach) with low or high adherence usage	DISCOUNTED INCREMENTAL COST OF PrEP STRATEGIES OVER 5‐YEAR TIME HORIZON Plan A Respective costs of Non‐targeting 10%; 30%; and 90% are $158,411,503; $398,568,822; and $1,127,434,311 Respective costs of Targeting 10%; 30%; and 90% are $89,432,361; $190,572,365; and $496,573,340 Plan B Respective costs of Non‐targeting 10%; 30%; and 90% are $ 52,137,608; $79,665,459; and $170,226,003 Respective costs of Targeting 10%; 30%; and 90% are $44,267,840; $55,056,540; and $89,865,774 Plan C Respective costs of Non‐targeting 10%; 30%; and 90% are $44,642,119; $57,173,233; and $102,714,188 Respective costs of Targeting 10%; 30%; and 90% are $41,082,391; $45,498,622; and $ 61,180,726	
Ying	SDC	As‐studied	INCREMENTAL COSTS (PER COUPLE) As‐studied (total clinical HIV treatment + PrEP): $1058, As‐studied SoC (HIV treatment without PrEP): $650, As‐studied PrEP: $408	No (Cost‐effectiveness only)
		MoH	INCREMENTAL COSTS (PER COUPLE) MoH total clinical: (HIV treatment + PrEP) $453, MoH standard of care (i.e. HIV treatment without PrEP): $361, MoH PREP: $92 MoH Assumptions showcasing reduction in as‐studied to MoH PrEP price As‐studied with PrEP with public‐sector staff salaries: $370, and reduced medication costs: $254, and fewer lab tests: $101, and PrEP task‐shifting: $92	
		MoH adds PrEP programme for all high‐risk SDC (i.e. when the HIV‐negative partner is aged < = 25 years and both partners are in the top 15th percentile in number of casual sexual partners). Scenario also assumes 40% baseline ART coverage, 80% of high‐risk couples are without CD4/VL criteria and 80% PrEP coverage among high‐risk couples	$219 million over 10 years	
Eakle	Sex worker	N/A	PrEP PER PERSON‐YEAR, Y1 Average: $126.60 (Johannesburg: $146.60, Pretoria: $106.60) COST PER VISIT Outreach contact visit—Average: $2.80 (Johannesburg: $3.00, Pretoria: $2.60) VCT Session—Average: $18.10 (Johannesburg: $21.2, Pretoria: $15.10) PrEP Enrolment Visit —Average: $34.70 (Johannesburg: $40.40, Pretoria: $29.00) PrEP Monitoring Visit —Average: $35.20 (Johannesburg: $37.40, Pretoria: $33.00) PrEP Refill Visit —Average: $6.80 (Johannesburg: $7.40, Pretoria: $6.20)	No
Roberts	AGYW	As‐implemented	Total annual programme cost: $204,253 Average cost of per client‐month of PrEP dispensed: $26.52 UNIT COST BY CLINICAL ACTIVITY PrEP screening —Total annual cost: $69,876; Total unit cost (variable + fixed): $2.91 PrEP initiation —Total annual cost: $80,525; Total unit cost (variable + fixed): $19.18 PrEP follow‐up ‐Total annual cost: $53,852; Total unit cost (variable + fixed): $12.16	Yes
		Service delivery modification: Postponed creatine testing to first follow‐up visit	Total annual cost: $188,932 Cost per client‐month of PrEP dispensed: $24.53	
		Service delivery modification: Prioritized delivery to clients at high risk for HIV infection	Total annual cost: $175,793 Cost per client‐month of PrEP dispensed: $31.88	
		As‐implemented scenario with public‐sector clinical staff salaries	Total annual cost: $199,613 Cost per client‐month of PrEP dispensed: $25.92	
		As‐implemented scenario with MOH supervision and public‐sector clinical staff salaries	Total annual cost: $138,609 Cost per client‐month of PrEP dispensed: $18.00	
		As‐implemented scenario with facility creatinine testing, MOH supervision and public‐sector clinical staff salaries	Total annual cost: $127,421 Cost per client‐month of PrEP dispensed: $16.54	
Irungu	SDC	As‐studied	ANNUAL COST OF DELIVERING INTEGRATED PrEP AND ART TO SDC Total cost: $757,483.58; Cost per couple: $1.454.87 ANNUAL INCREMENTAL COST OF ADDING PREP TO CURRENT ART PROGRAMME Total cost: $441,555.40; Cost per couple: $305.75	Yes
		Current care and PrEP with MoH costs	ANNUAL COST OF DELIVERING INTEGRATED PrEP AND ART TO SDC Total cost: $361,304.58; Cost per couple: $250.19 ANNUAL INCREMENTAL COST OF ADDING PREP TO CURRENT ART PROGRAMME Total cost: $125,338.15; Cost per couple: $86.79 per couple	
		Current care & PrEP costs (removing research costs)	ANNUAL COST OF DELIVERING INTEGRATED PrEP AND ART TO SDC Total cost: $962,032.84; Cost per couple: $66.16	
Hughes	SDC	SAFER	Individual strategy—PrEP: $1229 per couple Multiple strategies: ART‐VL + PrEP: $1709 per couple; PrEP + SW: $1659 per couple; PrEP + AVI: $1242 per couple	Yes
		High intensity (study‐level) with real‐world prices	Individual strategy—PrEP: $403 per couple Multiple strategies: ART‐VL + PrEP: $517 per couple; PrEP + SW: $771 per couple; PrEP + AVI: $408 per couple	
		Target intensity, incremental cost added to CP	Individual strategy—PrEP: $266 per couple Multiple strategies: ART‐VL + PrEP: $483 per couple; PrEP + SW: $563 per couple; PrEP + AVI: $291 per couple	
		Target intensity, incremental cost added to SOC	Individual strategy—PrEP: $88 per couple Multiple strategies: ART‐VL + PrEP: $166 per couple; PrEP + SW: $387 per couple; PrEP + AVI: $114 per couple	
Peebles	Other (see below)	N/A	FINANCIAL COSTS Total: $91,175; Cost per PrEP Client: $35.52; Cost per person‐month of PrEP: $10.31 ECONOMIC COSTS Total: $188,584; Cost per PrEP Client: $73.46; Cost per person‐month of PrEP: $21.32	No
Wanga	AGYW	POWER Study scenario	Estimated Total (variable + fixed) economic costs Annual cost: $44,933; Cost per client‐month of PrEP: $28.92 Estimated Total (variable + fixed) economic costs by visit type Initiation—Annual cost: $23,520; Cost per client‐month of PrEP: $47.09 Follow‐up—Annual cost: $20,896; Cost per client‐month of PrEP: $20.99	No
		MoH scenario	Estimated Total (variable + fixed) economic costs Annual cost: $22,566; Cost per client‐month of PrEP: $14.52 Estimated Total (variable + fixed) economic costs by visit type Initiation—Annual cost: $10,156; Cost per client‐month of PrEP: $20.33 Follow‐up—Annual cost: $11,997; Cost per client‐month of PrEP: $12.05	
		Scaled‐MoH scenario	Estimated Total (variable + fixed) economic costs Annual cost: $83,196; Cost per client‐month of PrEP: $10.88 Estimated Total (variable + fixed) economic costs by visit type Initiation—Annual cost: $38,352; Cost per client‐month of PrEP: $11.84 Follow‐up—Annual cost: $43,213; Cost per client‐month of PrEP: $9.81	
Hendrickson	AGYW	Integrated into DREAMS, including community sensitization and demand creation through short‐term mobilizers	Total recurrent cost per PrEP‐client per year: $320 Total average cost per PrEP‐client per year: $394 Total cost per person‐month: $33	No
	GP	Integration Model 1: Outreach using trained CHW to sensitize community about PrEP and refer interested people to nearest clinic	Total recurrent cost per PrEP‐client per year: $530 Total average cost per PrEP‐client per year: $760 Total cost per person‐month: $63	
		Integration Model 2: Site readiness through preliminary site assessments, trainings, community consultations and global technical assistance	Total recurrent cost per PrEP‐client per year: $381 Total average cost per PrEP‐client per year: $406 Total cost per person‐month: $34	
		Integration Model 3: Community HIV Epidemic Model of care, focused on community education, mobilization, PrEP sensitization, and training CHW on key population sensitivity and PrEP services	Total recurrent cost per PrEP‐client per year: $586 Total average cost per PrEP‐client per year: $659 Total cost per person‐month: $55	
	Other (FSW & MSM together)	Targeted community‐based demand creation with referrals to local facilities for PrEP initiation and follow‐up	Total recurrent cost per PrEP‐client per year: $350 Total average cost per PrEP‐client per year: $425 Total cost per person‐month: $35	
Okal	AGYW	N/A	Costs of delivering dreams interventions Urban Total: $215,440; Cost of providing DREAMS interventions to 1 AGYW: $67 Peri‐urban Total: $408,884; Cost of providing DREAMS interventions to 1 AGYW: $129	Yes
Mudimu	AGYW	As‐implemented	Incremental cost of community‐based prep provision Standard of Care Annual cost: $135,314; Cost per person month of PrEP: $105.74 Club Annual cost: $135,143.42; Cost per person month of PrEP: $105.61 Individual Annual cost: $135,791.40; Cost per person month of PrEP: $106.09	No
		DoH scenario	Incremental cost of community‐based prep provision Standard of Care Annual cost: $70,944.44; Cost per person month of PrEP: $55.46 Club Annual cost: $70,811.93; Cost per person month of PrEP: $55.32 Individual Annual cost: $71,227.59; Cost per person month of PrEP: $55.65	
		Scaled‐DoH scenario	Incremental cost of community‐based prep provision Standard of Care Annual cost: $142,342.77; Cost per person month of PrEP $13.99 Club Annual cost: $144,881.73; Cost per person month of PrEP: $15.48 Individual Annual cost: $133,822.61; Cost per person month of PrEP: $26.40	
Mangenah	AGYW	N/A	Cost per person‐year of receiving oral PrEP: $839 Average costs by visit type Initiation: $240 per client initiated, Month 3 follow‐up: $434 per client continued to 3 months, Month 6 follow‐up: $844 per client continued to 6 months	Yes
	Men	N/A	Cost per person‐year of receiving oral PrEP: $1219 Average costs by visit type Initiation: $215 per client initiated, Month 3 follow‐up: $712 per client continued to 3 months, Month 6 follow‐up: $1363 per client continued to 6 months	
	Women	N/A	Cost per person‐year of receiving oral PrEP: $857 Average costs by visit type Initiation: $243 per client initiated, Month 3 follow‐up: $480 per client continued to 3 months, Month 6 follow‐up: $828 per client continued to 6 months	

Abbreviations: AGYW, adolescent girls and young women; ART‐VL, antiretroviral therapy with frequent viral load testing; AVI, manual artificial vaginal insemination; CP, Current Practice; DoH, Department of Health; FSW, female sex workers; GP, general population; MoH, Ministry of Health; MSM, men who have sex with men; PrEP, pre‐exposure prophylaxis; SDC, sero‐different couples; SOC, Standard of Care; SW, semen washing; TDF/FTC, tenofovir disoproxil and emtricitabine.

**Table 9:** Cost‐effectiveness outcomes of primary costing studies

Author	Population(s)	Scenarios	Findings	Sensitivity analysis performed
Suraratdecha	MSM	Providing PrEP to only high‐risk MSM (defined as having engaged in condomless sex with casual or known HIV‐positive partners) versus all MSM, regardless of risk	Cost‐Effectiveness: PrEP provided to High‐risk MSM: $3.99 (M) lifetime treatment costs averted PrEP provided to All MSM: $9.84 (M) lifetime treatment costs averted ICER OVER 5‐YEAR TIME HORIZON PrEP provided to High‐risk MSM: $4836 per DALY averted; $68,468 per HIV infection averted PrEP provided to All MSM: $7089 per DALY averted; $100,367 per HIV infection averted	Yes
Wong	MSM	Basecase with 10%, 30% and 90% coverage of PrEP involving low‐risk and high‐risk MSM (i.e. non‐targeting approach) with low or high adherence usage and high‐risk MSM only (i.e. targeting approach) with low or high adherence usage Plans (apply to both scenarios) Plan A: PrEP priced at the market rate: $7880/year Plan B: PrEP priced at the generic rate: $519/year Plan C: PrEP is free	DISCOUNTED INCREMENTAL COST‐EFFECTIVENESS (INCREMENTAL $/QALY GAINED) OF PrEP STRATEGIES OVER 5‐YEAR TIME HORIZON Plan A Respective incremental cost‐effectiveness of Non‐targeting 10%; 30%; and 90% are $1,842,204; $1,745,524; and $2,115,619 Respective incremental cost‐effectiveness of Targeting 10%; 30%; and 90% are $2,162,072; $1,583,136; and $1,642,874 Plan B Respective incremental cost‐effectiveness of Non‐targeting 10%; 30%; and 90% are $258,064; $243,483; and $298,518 Respective incremental cost‐effectiveness of Targeting 10%; 30%; and 90% are $306,779; $219,862; and $228,540 Plan C Respective incremental cost‐effectiveness of Non‐targeting 10%; 30%; and 90% are $146,335; $137,545; and $170,358 Respective incremental cost‐effectiveness of Targeting 10%; 30%; and 90% are $175,926; $123,710; and $128,788	Yes
		Test‐and‐Treat included a high rate of diagnosis and treatment initiation (minimum 90% from 2017) with 10%, 30% and 90% coverage of PrEP involving low‐risk and high‐risk MSM (i.e. non‐targeting approach) with low or high adherence usage and high‐risk MSM only (i.e. targeting approach) with low or high adherence usage	DISCOUNTED INCREMENTAL COST‐EFFECTIVENESS (INCREMENTAL $/QALY GAINED) OF PrEP STRATEGIES OVER 5‐YEAR TIME HORIZON Plan A Respective incremental cost‐effectiveness of Non‐targeting 10%; 30%; and 90% are $929,125; $1,345,390; and $1,985,645 Respective incremental cost‐effectiveness of Targeting 10%; 30%; and 90% are $668,940; $956,132; and $1,366,821 Plan B Respective incremental cost‐effectiveness of Non‐targeting 10%; 30%; and 90% are $305,830; $268,915; and $299,803 Respective incremental cost‐effectiveness of Targeting 10%; 30%; and 90% are $331,116; $276,227; and $247,356 Plan C Respective incremental cost‐effectiveness of Non‐targeting 10%; 30%; and 90% are $261,863; $192,991; and $180,901 Respective incremental cost‐effectiveness of Targeting 10%; 30%; and 90% are $307,290; $228,274; and $168,400	
Ying	SDC	MoH adds PrEP programme for all high‐risk SDC (i.e. when the HIV‐negative partner is aged < = 25 years and both partners are in the top 15th percentile in number of casual sexual partners). This scenario also assumes 40% baseline ART coverage, 80% of high‐risk couples are without CD4/VL criteria and 80% PrEP coverage among high‐risk couples	ICER OVER 10‐YEAR HORIZON $1340 per HIV infection averted $5354 per DALYs averted	Yes

Abbreviations: ART, antiretroviral therapy; DALYs, disability‐adjusted life year; ICER, incremental cost‐effectiveness ratio; MSM, men who have sex with men; PrEP, pre‐exposure prophylaxis; QALY, quality‐adjusted life year; SDC, sero‐different couples; VL, viral load.

### 3.11 Implementation domains regarding long‐acting PrEP

None of the 10 modelled studies of LAP included costs on target setting, supply chain logistics and management, health information systems, operations research and implementation science, or above‐site domains (Table 4). Further, only PrEP commodities (*N* = 9) and human resources (*N* = 5) domains were reported >50% of the time. The other domains were reported at frequencies of 2 and 1 for the five injectable LAP studies and four studies on the PrEP ring (Table 4).

## 4 DISCUSSION

This scoping review synthesized national implementation plans from four costed rollout plans (CRPs) operationalizing oral PrEP from Kenya, South Africa, Zambia, and Zimbabwe, and the FP CIP template. Few CRPs have been developed to support daily oral PrEP, and no plans included LAPs. The AVAC tracker showed that over 20 SSA countries approved PrEP, yet only four implementation plans were found. Interestingly, these four countries represent over 50% of SSA PrEP initiations between 2016 and 2022 [19]. While all countries have fallen short of HIV prevention benchmarks, the crude correlation is suggestive that CRPs reflect country‐level and ‐led discussions about systematic planning of implementation, an indication of national commitment and political will, which may contribute to greater PrEP achievement.

Our CRP review identified 15 domains as necessary inputs and activities. Six of the 15 domains were reported in all plans reviewed; however, we cannot conclude that they are more important than domains not consistently included since plans were not based on rubrics or frameworks. We observed variations in the naming of the implementation domains and incorporated activities or inputs. Also, CRP domains lacked any prioritized order or hierarchy, highlighting the need for a framework for developing CRPs and a consensus process for composing activities and inputs. We surmised that all domains were method agnostic and relevant for implementing LAP (Table 4). CRPs will be important because of LAPs complexity of choice, variation in logistics ,and likely cost.

Further, most (80%) of the 66 studies published between January 2010 and 30 June 2022 were secondary data or modelled evaluations, and few (19.6%) reported primary costing. The 15 implementation domains were included inconsistently. None of the 13 primary costing studies included all 15 domains, but most included: PrEP commodities, human resources, indirect cost, and other associated commodities. Few primary costing studies included policy and planning, supply chain and logistics, or above‐site costs, which include technical assistance and domains that were present in all implementation plans. Modelled or cost‐effectiveness studies, many of which applied a guideline costing approach to inform policy and planning, included fewer domains than primary costing studies. We observed that most studies did not meet GHCC criteria, including variable cost and cost‐effectiveness outcomes and units of measure. The lower quality, inconsistencies, and lack of transparency in cost and cost‐effectiveness studies have been raised by others, but to our knowledge, this is the first study to examine the cost and cost‐effectiveness literature against real‐world operational plans and assess implementation inputs and activities with such specificity [32].

The unsystematic delivery of HIV services has been highlighted as a persistent structural impediment that diminishes the impact of HIV prevention interventions [1, 3]. LAPs will undoubtedly increase the complexity of HIV prevention delivery at all levels of the health system. LAPs vary in reported efficacy and effectiveness, user preferences, laboratory requirements to initiate and sustain use, supply chain needs to deliver a safe and effective product, and monitoring and evaluation for continuous quality improvement, among others [54, 55, 56, 57]. Terris‐Prestholt et al. previously stated that plans outlining systematic introduction and delivery of PrEP, including impact on efficiency, uptake, and equity, are needed to manage scarce human and financial resources. We need user‐tailored messaging and optimized delivery channels to reach high‐need groups given resource constraints [58]. CIPs, multi‐year actionable roadmaps designed to help governments achieve their FP goals by facilitating systematic implementation, scale‐up, and financing, have facilitated many programmatic achievements owing to streamlined planning, implementation and stakeholder consultation, and systematic costing [41, 59]. Amid the scarcity of costed plans for operationalizing HIV PrEP delivery [25, 26, 27, 28], CIPs and national costed HIV plans serve as a model for LAP rollout in countries and settings not currently applying this approach.

As noted above, plans did not mention a rubric guiding which implementation domains to include and the rationale for including some domains over others. A lack of specificity on what is needed to implement LAP will further stymy implementation given the current concerns about the cost of LAP. LAP options are being introduced in the context of static prevention funding. The costs of LAPs are considered a major determinant and threat to their implementation [60]. Stakeholders debate the lack of transparency in injectable PrEP and alternative pricing. For instance—stating that success will require cabotegravir to be offered at an affordable price is all the more concerning when injectable PrEP's threshold for cost‐effectiveness in South Africa is <$100 per person‐year [61, 62]. Moreover, due to COVID‐19 and ongoing global economic instability, some policymakers and other stakeholders call for strategies to improve efficiencies in HIV investments [63]. Understanding the current oral PrEP and LAP rollout, full and incremental costs can inform programme efficiencies and financing innovations. This dearth of primary cost data across the full implementation process, concern about transparent LAP pricing, and nascent understanding of the evolving implementation landscape with the introduction of LAPs elevate the importance of systematic real‐world delivery accompanied by primary cost data.

We described how implementation domains included in studies were operationalized in the current context of PrEP delivery. This serial scoping review identified and addressed several literature gaps. First, our rapid review of implementation plans revealed: (1) the rarity of national costed PrEP rollout plans despite expanded PrEP delivery in resource‐constrained settings; and (2) the variability of plans’ composition and costed domains. Additionally, our scoping review of primary costing and model‐based evaluations highlighted (1) a preliminary understanding of real‐world PrEP costs due to a preponderance of model‐based studies; (2) primary costed studies only explored 12 of the 15 implementation domains, overlooking target setting, health information systems, and implementation science research; and (3) costing units and assumptions varied greatly across primary costing studies, precluding comparison. Together, our reviews highlight the need to further refine and prioritize: (1) content areas for PrEP implementation plans in LMICs; and (2) templates and resources to systematically develop CRPs for approved LAP methods.

No study costed all implementation domains. PrEP costs will need to be more appropriately estimated on a case‐by‐case basis according to the scope of required activities in each country. For instance, how will countries establish targets for PrEP overall and disaggregated by PrEP methods, including LAP? What technical resources will be needed to estimate initial targets and use various data to update targets as implementation progresses? Health information systems will need to be updated to include multiple methods with different frequencies of use, routes of administration, and means of monitoring. Implementation research is also critical to identify ways that complex interventions could be bundled together and identify implementation strategies that facilitate uptake and effective use of LAP. Investments in supply chain and logistics may also require consideration of the unique needs of injectable Cab PrEP or the push to de‐medicalize the PrEP ring. Many studies that cost human resources included costs for training and supportive supervision, both of which will be critical for LAP. Implementers grapple with setting metrics to monitor and evaluate LAP use in programmes, requiring alterations in the health information systems [64]. PrEP metrics need to measure LAP delivery and uptake in service points like FP, where LAP could be integrated [65]. However, no studies accounted for costs associated with updating information systems. Since only 20% of PrEP studies included primary cost data collection, this suggests that most economic evaluations use historical estimates that may no longer represent the current implementation landscape.

PrEP commodities and human resources were the domains most included in modelled evaluations. As with primary cost studies, activities like implementation research to inform decision‐making, target setting, awareness raising, technical assistance, and health information system strengthening—vital and catalytic investments in the early phase of introducing new products into the public health system—were not included. Our findings corroborate other reviews examining PrEP cost and cost‐effectiveness in mathematical modelling studies [23, 66, 67, 68, 69, 70, 71, 72, 73, 74, 75, 76, 77, 78, 79]. Case et al. highlighted in a 2019 review the outdated assumptions in the modelling literature, the lack of “real‐world costing” and the limitation of the modelling studies to include programme implementation, among other factors [23]. To our knowledge, our paper is the first analysis that examined the intersection of implementation plans and costing or economic evaluations explicitly.

The wide variation in cost outcomes poses a synthesis challenge identified in this review and previously [32]. Standardized costing instruments should be created, including guidance on costing attributes for inclusion and the process of costing PrEP. Appropriate costing units can help establish higher‐quality, consistent cost data to inform planning for LAP. One example can be found on Prepwatch [80]. Around 2016, we observed an increased frequency and diversity of PrEP methods in the cost and economic literature, 1 year after daily oral PrEP was recommended for all by the WHO [14]. With WHO's recommendation, country‐level approval and the imminent introduction of LAP, priority should be placed on primary costing studies of LAPs that align with real‐world implementation needs [81]. This is an important time for costing experts and decision‐makers to ensure that standards are in place for the comprehensive primary costing of LAP. Health economic studies are instrumental to policy and programme planning and should broaden the scope of costed activities to better reflect the real‐world implementation, as also noted by Torres‐Rueda et al. [35].

### 4.1 Limitations

Our scoping review focused on peer‐reviewed English‐language literature only. We tried to overcome this limitation by utilizing nine electronic databases, including several databases that index work in SSA, which allowed us to cross‐reference extensively. Additionally, we reached out through professional networks to identify grey literature. This effort yielded additional peer‐reviewed literature through collaborators (authors FTP, FB and STR). We identified implementation domains through a rapid review of four country plans and conducted the thematic analysis inductively. These domains have not been vetted beyond our study team. Other countries may have plans that were not publicly or easily accessed. As a result, we may classify activities differently. Further work is needed to develop a stakeholder‐informed consensus template for key domains to consider when planning implementation. Implementation frameworks (e.g. AGREE‐II or GLIA) may provide some additional structure that is validated, but based on our assessment, these frameworks would need to be modified to fulfil the purpose of the implementation plan review and evaluation [82, 83]. The GHCC reference case provided methodological principles for evaluating the studies. However, operationalizing the components of the principles in this evaluation revealed that the principles are complex and multidimensional, and the categories are not often mutually exclusive. These factors make it difficult to apply the principles quantitatively.

We note the significant lack of primary data collection related to the cost of delivering PrEP (particularly LAP). For those not reliant on primary data collection, or when the report was unclear, the research team could not analyse how unit cost data were obtained (e.g. budgetary figures, modelling cost estimates, imputation from neighbouring countries, etc.). Further analysis of these studies should allow for a more in‐depth review of what figures are currently being used and their comparability. As a result, the studies differed in the scope of interventions offered, and in that sense, mixed‐cost data are not comparable. As an example, some PrEP interventions spend significantly on client retention, while other interventions do not. Meaningful conclusions can, therefore, not be drawn from head‐to‐head comparisons of studies.

This synthesis included studies with different analytic approaches, populations, and assumptions that will result in very different cost estimates. For instance, some studies focused on financial costs, while others focused on economic costs. As a result, it needed to be clarified if or how studies dealt with costing in‐kind resources. Differences in interventions (PrEP as part of an integrated HIV prevention or other health programmes), staff time and burden (time‐and‐motion analysis vs. provider interviews), and targeting (details about specific populations vs. cost estimates per person reached) also existed. The inherent heterogeneity of these studies is both a limitation and a strength in ascertaining assumptions.

## 5 CONCLUSIONS

The successful integration and scale‐up of new LAP methods into existing service delivery will depend on robust implementation built on sound logistical and financial planning. Based on these observations, we recommend: (1) a framework and tools to support countries in developing CRPs for LAP; (2) a process to ensure that there is global consensus on the composition of domains, defining activities and inputs, and a process for developing costed plans and determining essential and optional components; (3) further examination of implementation considerations for LAP and for supporting a method mix of biomedical HIV prevention; and (4) we echo the call for improved quality, consistency, and transparency in cost and cost‐effectiveness studies developed to inform national planning.

## COMPETING INTERESTS

The authors declare no competing interests.

## AUTHORS’ CONTRIBUTIONS

DC, CJH and SF conceptualized the manuscript. EG and NVT performed independent title and abstract screening for eligibility and initial full‐text review articles. DC, SF and CJH independently reviewed all articles with discrepant reviews and a subset of initially concordant reviews. DC, SF, CJH, NVT, JW and KKu extracted relevant data from the included studies into REDCap. DC, SF and CJH cross‐validated data entry. FB, FT‐P and STR performed an independent review that supplemented the search and review. DC and CJH prepared the first draft of the manuscript. All authors edited and commented on the drafts of the manuscript. All authors have read and approved the final manuscript.

## FUNDING

This work was made possible by the generous support of the American people through the U.S. President's Emergency Plan for AIDS Relief (PEPFAR) and the U.S. Agency for International Development (USAID) cooperative agreements 7200AA21CA00011 and AID‐OAA‐A‐15‐00045, and through the National Institute of Allergy and Infectious Diseases of the National Institutes of Health under Award Number T32AI114398 (CJH), K23AI150378 (JZ) and UM1AI069470 (MES).

## DISCLAIMER

The content is solely the responsibility of the authors. It does not necessarily reflect the views of PEPFAR, USAID, NIH or the U.S. Government and also does not represent the official views of the United States Agency for International Development, the National Institutes of Health or the Global Fund for AIDS, Tuberculosis and Malaria (GFATM).

## REFERENCES OF INCLUDED STUDIES

**Table:** 

Author	Year of publication	Reference
Ying	2015	[48]
Eakle	2017	[49]
Suraratdecha	2018	[50]
Wong	2018	[84]
Irungu	2019	[43]
Roberts	2019	[52]
Hughes	2020	[45]
Peebles	2021	[47]
Hendrickson	2021	[46]
Wanga	2021	[53]
Mudimu	2022	[42]
Mangenah	2022	[44]
Okal	2022	[51]
Smith	2016	[85]
Stover	2016	[86]
Walensky	2016	[87]
Glaubius	2016	[88]
Quaife	2018	[89]
van Vliet	2019	[90]
Glaubius	2019	[91]
Reidy	2019	[92]
Vogelzang	2020	[93]
Adeoti	2021	[94]
Pretorius	2010	[95]
Hallett	2011	[96]
Gomez	2012	[97]
Long	2013	[98]
Cremin	2013	[99]
Nichols	2013	[100]
Verguet	2013	[101]
Stover	2014	[102]
Nichols	2014	[103]
Anderson	2014	[104]
Alistar	2014	[105]
Alistar	2014	[106]
Cremin	2015	[107]
Jewell	2015	[108]
Cremin	2015	[109]
Mitchell	2015	[110]
Price	2016	[111]
Moodley	2016	[112]
Meyer‐Rath	2017	[113]
Chiu	2017	[114]
Cremin	2017	[115]
Akudibillah	2017	[116]
Alsallaq	2017	[117]
Anderson	2018	[118]
Li	2018	[119]
Luz	2018	[120]
Stopard	2019	[121]
Zhang	2019	[122]
Bórquez	2019	[123]
Selinger	2019	[124]
Hu	2019	[125]
Grant	2020	[126]
Kazemian	2020	[127]
Pretorius	2020	[128]
Jamieson	2020	[129]
Kazemian	2020	[130]
Wu	2021	[131]
Phillips	2021	[132]
Ten Brink	2022	[133]
Kripke	2022	[134]
Jin	2022	[135]
Phillips	2022	[136]
Ghayoori	2022	[137]

## Supporting information


**Supporting Information 1**: Timeline and populations of World Health Organization Guidance for Biomedical HIV Prevention Strategies.
**Supporting Information 2**: Preferred Reporting Items for Systematic reviews and Meta‐Analyses extension for Scoping Reviews (PRISMA‐ScR) Checklist
**Supporting Information 3**: Search queries used for electronic database searches
**Supporting Information 4A**: Frequency of Populations Costed within Included Studies
**Supporting Information 4B**: Frequency of Populations Represented within Included Studies
**Supporting Information 5A**: Frequency of Geographies with Regional, National, or Sub‐National Costing Data within Included Studies
**Supporting Information 5B**: Frequency of Countries and Regions Represented within Included Studies
**Supporting Information 6**: Economic Analysis Features and Analytic Approaches of Secondary Costing or Modeling Studies (Inclusive of bnAbs and HIV Vaccine columns)

## Data Availability

Data are made available upon request to the primary author.
